# Characterization of a Novel Diarrheagenic Strain of *Proteus mirabilis* Associated With Food Poisoning in China

**DOI:** 10.3389/fmicb.2019.02810

**Published:** 2019-12-12

**Authors:** Zelong Gong, Xiaolu Shi, Fang Bai, Xiaolong He, Hanyun Zhang, Yubin Li, Yu Wan, Yiman Lin, Yaqun Qiu, Qiongcheng Chen, Qinghua Hu, Hong Cao

**Affiliations:** ^1^Department of Microbiology, Guangdong Provincial Key Laboratory of Tropical Diseases, School of Public Health, Southern Medical University, Guangzhou, China; ^2^Shenzhen Major Infectious Disease Control Key Laboratory, Shenzhen Center for Disease Control and Prevention, Shenzhen, China

**Keywords:** *Proteus mirabilis*, diarrhea, cell and mouse models, food poisoning, type IV secretion system

## Abstract

*Proteus mirabilis* is commonly considered to be an opportunistic pathogen causing urinary tract infections (UTIs) in humans. However, some strains of *P. mirabilis* were found to be associated with food poisoning outbreaks, with the pathogenic mechanism still unclear. In our study, we described a novel strain of *P. mirabilis* C02011 isolated from patients’ specimens in a food poisoning in China. In order to determine its gastrointestinal pathogenicity, experiments were performed to compare *P. mirabilis* B02005 strain (isolated from healthy people) and *P. mirabilis* American Type Culture Collection (ATCC) 29906 strain both *in vitro* [Caco-2 cells: bacterial adhesion and invasion assays, Giemsa staining, and transmission electron microscopy (TEM)] and *in vivo* [BALB/c mouse model: fecal character, colon injury, histological examination, immunochemistry, and western blotting (WB)]. According to the results, C02011 strain exhibited almost identical characteristics with B02005 strain in bacterial appearance and proliferation. *In vitro*, Caco-2 cells were infected with *P. mirabilis* C02011, B02005, and *P. mirabilis* ATCC 29906 strains. After that, Giemsa staining and TEM were used for observing the infection process of C02011 strain. Meanwhile, the adhesive abilities of different strains were rated as follows: *P. mirabilis* B02005 > *P. mirabilis* C02011 > *P. mirabilis* ATCC 29906 (*P* < 0.01). Invasive abilities of different strains were rated as follows: *P. mirabilis* C02011 > *P. mirabilis* B02005 > *P. mirabilis* ATCC 29906 (*P* < 0.01). *In vivo*, BALB/c mice were infected with *P. mirabilis* C02011 and B02005 strains. C02011 strain shows more virulence than B02005 strain in terms of the following indicators: (1) feces water content and fecal character; (2) colon length of mice; (3) histological examination on mouse intestine tissues; (4) ELISA for detecting TNF-α level in the colon; and (5) WB and immunohistochemistry (IHC) for detecting occludin protein expression in the colon. On the basis of these results, we firstly validated that the novel strain of *P. mirabilis* C02011 shows more gastrointestinal pathogenicity than the other strains isolated from a healthy individual. In addition, type IV secretion system (T4SS) was preliminarily confirmed to play an important role in the pathogenesis of diarrheal *P. mirabilis* isolated from the food poisoning incident.

## Introduction

*Proteus mirabilis* is a gram-negative bacterium with flagella around the body, which is widely found in water, soil, and human intestinal environments. Commonly, *P. mirabilis* is harmless to human health. Sometimes, *P. mirabilis* is considered as the causative pathogen in hospital cross-infection associated with urinary catheter (O’Hara et al., 2000; Hola et al., 2012). However, some novel strains have been reported to pose risks to human health and may cause serious disease in patients (Rozalski et al., 1997; Wang et al., 2010). These strains isolated from hospitals have been identified as virulent pathogens in patients suffering from human sepsis, food poisoning, peritonitis, and meningitis. In recent years, more and more food poisoning cases associated with *P. mirabilis* had been reported in China. In these food poisoning incidents, clinical symptoms of the patients infected with *P. mirabilis* included abdominal pain, diarrhea, nausea, and dizziness (Wang et al., 2010; Shi et al., 2014, 2016). For example, 256 students in a middle school were diagnosed with food poisoning of *P. mirabilis* in Zhejiang Province in 1998 (Liu and Lu, 2000); 34 people were diagnosed with food poisoning of *P. mirabilis* in Guangxi Province in 2006 (Liang et al., 2007); and four people were identified to have food poisoning of *P. mirabilis* in Beijing in 2013 (Huo et al., 2014). According to a surveillance report of bacterial food poisoning issued by Datong Food and Drug Inspection and Testing Center, food poisoning incidents related to *P. mirabilis* accounted for 3.61% in the food poisoning incidents reported in Datong from 2016 to 2017 (Shanxi Province, China).

In addition, food poisoning incidents associated with *P. mirabilis* have been reported worldwide for a long time, and more researchers began exploring the gastrointestinal virulence and rapid detection techniques of *P. mirabilis*. In the United Kingdom, a food poisoning incident related to *Proteus* involving eight cases was reported in Bristol Royal Infirmary; the contaminated food were brawn and tomato puree (Cooper et al., 2005). In Turkey, Reyhan and his team have explored the antimicrobial effect of garlic on foodborne pathogens including *Escherichia coli*, *Salmonella enteritidis*, and *P. mirabilis* AUFE 43566 (Irkin and Korukluoglu, 2009). *Encyclopedia of Food Microbiology* (second edition) mentioned that *P. mirabilis* may play a role in food spoilage and as enteropathogens (Kushwaha et al., 2014). *Bad Bug Book* (“Handbook of foodborne pathogenic microorganisms and natural toxins”), issued by the Food and Drug Administration [FDA] (2012), mentions that “With regard to foods, *Proteus* could metabolize amino acids found in meats to produce compounds that may cause putrefaction. In fish, such as tuna, *Proteus* is considered a histamine-producing microbe and under such circumstances could generate scombroid poisoning.” Epidemiological investigations indicated that meat products, bean products, fish, and cold dishes are commonly associated with food poisoning related to *P. mirabilis* (Wang et al., 2010; Cao et al., 2011; Jiang et al., 2017). Hence, *P. mirabilis* may pose a relatively great threat to food safety and public health.

Nowadays, the pathogenic mechanism of diarrheal *P. mirabilis* is still unclear. It is essential and meaningful to explore the gastrointestinal pathogenicity of the *P. mirabilis*, which may cause human diarrhea. Till now, few published studies have explained the pathogenic mechanism of diarrheal *P. mirabilis*. In our study, two strains of *P. mirabilis* have been isolated from clinical specimens: *P. mirabilis* C02011 strain was isolated from patients’ vomitus and feces specimens in a food poisoning in Shenzhen; and *P. mirabilis* B02005 strain was isolated from feces specimen of healthy people. Our preliminary works (Shi et al., 2014, 2016) have confirmed our speculation that several virulence genes (*urease* gene, *hemolysin* gene, and *metalloproteinase* gene) were detected in *P. mirabilis* C02011 by using whole genome sequencing and phylogenetic analysis, which were not found in *P. mirabilis* B02005 strain (Shi et al., 2016). Notably, type IV secretion system (T4SS), which is commonly found in pathogenic bacteria such as *Klebsiella pneumoniae* and *Helicobacter pylori* (Wang et al., 2016; Medrano and Bell, 2017), has also been identified in *P. mirabilis* C02011 (Shi et al., 2016).

From the above, we speculated that *P. mirabilis* C02011 was a novel strain with more virulence, which may possibly be associated with diarrhea in susceptible population, whereas *P. mirabilis* B02005 with less virulence may not cause diarrhea among the susceptible population. In order to determine the gastrointestinal pathogenicity of *P. mirabilis* C02011, we built an experimental model both *in vitro* and *in vivo*. In addition, T4SS was preliminarily confirmed to play an important role in the pathogenesis of diarrheal *P. mirabilis* C02011 *in vitro* in the study, so as to lay a foundation work for the following researches on exploring its mechanism.

## Materials and Methods

### Bacterial Strains

#### Ethics Statement on Bacterial Isolation

*Proteus mirabilis* C02011 strain was isolated, detected, and stored in Shenzhen Center for Disease Control and Prevention (SZCDC) following the standards of GB/T4789-2003 (GB/T4789.1, 17, 22, 23, and 28) and WS/T9-1996. As a reference lab and pathogenic microorganisms’ repository in local region, SZCDC is the institution that is responsible legally for investigating the pathogen contamination and food poisoning. Hence, ethics approval for the isolation of strains was not provided in the study. *P. mirabilis* B02005 was isolated from a healthy individual; informed consent for feces collection was agreed upon by the volunteer.

#### Bacterial Strains, Isolation, and Culture

On June 2002, a food poisoning occurred in Luohu District, Shenzhen, China. Five patients with clinical syndromes of nausea, vomiting, abdominal pain, watery diarrhea, and dizziness were sent to the hospital for treatment. Meanwhile, the food poisoning case was reported to SZCDC for further investigation. According to the investigation of SZCDC, *P. mirabilis* C02011 was isolated from the residue food (cold dishes), vomited matter, excrement, and anal swab of the patients. Results of pulsed-field gel electrophoresis (PFGE) indicated that *P. mirabilis* C02011 was associated with this food poisoning case (Shi et al., 2016).

In our study, food samples, stool specimens, and vomit specimens collected from the patients were sent to the laboratory for examination within 24 h. Based on the phenotypic properties of morphology and biochemical features (WS/T 9-1996: Diagnostic criterion and principles of food poisoning induced by *P. mirabilis*; GB/T4789-2003: National criterion for food microbiological examination), *P. mirabilis* was successfully identified from the food poisoning incident (Shi et al., 2016). Meanwhile, Sensititre Auto-reader (Sensititre, United Kingdom) system was used to detect the bacteria. Results consistently showed that the suspicious colonies isolated from the patients’ specimens were *P. mirabilis*.

Strains involved in the study were isolated from various sources in SZCDC (Shi et al., 2016). *P. mirabilis* C02011 strain was isolated from patients’ specimens in a food poisoning in Shenzhen, whereas *P. mirabilis* B02005 was isolated from the anal swab of a healthy individual with no clinical symptoms. *P. mirabilis* was prepared on the logarithmic phase for the experiments by inoculating in brain heart infusion (BHI) medium with a single colony grown on MacConkey (MAC) agar plates at 37°C with shaking (160 rpm) for 16 h.

#### Growth Curve of Bacterial Strains

Strains of *P. mirabilis* were inoculated on MAC agar plates at 37°C for 16 h. Then, the single colony on MAC was selected and inoculated in sterilized BHI medium. Densicheck^®^ (Bio Mérieux, France) was used for detecting the bacteria density by measuring the turbidity at different times after inoculating at 37°C with shaking (160 rpm). Finally, growth curves of *P. mirabilis* were obtained based on turbidities.

#### Constructing the Mutant Strain With *T4SS* Genes Deletion

Primers were designed and synthesized according to the DNA sequences besides *T4SS* genes in *P. mirabilis* C02011. A PCR technique was used for amplifying two DNA sequences (A and B fragments) on both sides of the *T4SS* genes. Then, A and B fragments were combined to form pGEM-T easy vector and connected together by the same *Bam*HI loci. After that, the vector was subcloned into suicide plasmid pCVD442. The suicide recombinant plasmid (Targeting Vector) pCVD442-ΔT4SS was constructed and electro-transformed into *E. coli* SMl0 λpir by conjugative transduction. The suicide plasmid pCVD442-ΔT4SS was transformed from SMl0 λpir to *P. mirabilis* C02011. Finally, strains with *T4SS* genes deletion were screened by ampicillin sensitivity test and PCR (see Supplementary Appendix 2 for technical flowchart).

### Cell Assays *in vitro*

#### Cell Cultures and Reagents

Human colorectal cancer HT-29 cells (HT-29), human colon cancer LoVo cells (LoVo), and human cloning colon adenocarcinoma Caco-2 cells (Caco-2) were obtained from the Cell Bank of Chinese Academy of Sciences. Cells were cultured at 37°C in a 5% CO_2_-humidified incubator in 90% Dulbecco’s modified Eagle medium (DMEM) (*Gibco*, United States) containing 10% fetal bovine serum (FBS) (*Gibco*, United States), and 1% non-essential amino acid (NEAA) (Sigma, Japan). Phosphate-buffered saline (PBS, pH = 7.4) were obtained from Shanghai Double Spiral Biotech Co., Ltd. Giemsa stain solution was bought from Zhuhai Besso Biotech Co., Ltd. Other chemical reagents were of the highest grade available commercially.

#### Adhesion and Invasion Assays

Adhesion and invasion assays on *P. mirabilis* were performed following a standard method (Mathoera et al., 2002; Yamada et al., 2006). Bacteria were prepared at a concentration of 10^8^ CFU/ml. Cells were infected with *P. mirabilis* for 2 h with multiplicity of infection (MOI) = 100 at 37°C in 5% CO_2_. After that, infected cells were washed three times with PBS. Adhesion of *P. mirabilis* was measured as the number of bacteria adhering to host cell. All experiments were performed in triplicate. Invasion assay (Yamada et al., 2006) on *P. mirabilis* was also performed in our study. As mentioned above, cells were infected with *P. mirabilis* for 2 h and then washed three times with PBS and covered with DMEM containing gentamicin (200 μg/ml) to kill extracellular bacteria. Afterward, invasion of *P. mirabilis* was measured as the number of bacteria inside the body of host cells.

#### Giemsa Stain

After being infected with *P. mirabilis*, cells were washed three times with PBS, fixed by the addition of methanol for 10 min, and stained with Giemsa stain for 15 min (Yamada et al., 2006). The stained cells on the slides were washed with tap water mildly, mounted with neutral resin after drying, and observed under an optical microscope (Axio Imager M2) (×200 magnification).

#### Preparation of Samples for Transmission Electron Microscopy

Caco-2 cells were prepared as described above in the invasion assay. Then, infected cells were fixed with 2.5% glutaraldehyde in 0.1 mol/L of sodium cacodylate buffer. After that, fixed cells were cut into sections and then washed with 0.1 mol/L of sodium cacodylate, dehydrated in ethanol, and finally embedded in Agar 100 resin. Sections were mounted on copper grids, stained with uranyl acetate and lead citrate, and examined using transmission electron microscopy (TEM) (Georgiev et al., 2017).

### Animal Assays *in vivo*

#### Ethics Statement

Animal experiments were approved by the Animal Care Committee of Southern Medical University (Guangzhou, China). Six-week-old BALB/c mice were obtained from Animal Experimental Center of Southern Medical University. All supplements including food, water, and other nutrients were autoclaved; and animals were kept in the animal facilities. The protocol was approved by the Animal Care Committee of Southern Medical University (protocol number L2015027). The experimental process that involved infectious pathogen was conducted in biosafety level 2 laboratory. All surgeries were performed under anesthesia with ketamine and lidocaine, and utmost efforts were taken to minimize suffering.

#### Animals and Experimental Design

Forty-five mice were randomly divided into three groups (C02011 group, B02005 group, and control group). All mice were given sterile water containing streptomycin (SM) (5 g/L) for 3 days (Suar et al., 2006; Elder et al., 2016). One day before infection, SM water was replaced with SM-free water (Suar et al., 2006). After intestinal colonization was reduced, mice in different groups were gavaged with *P. mirabilis* C02011 (2.5 × 10^8^ CFU/mouse), *P. mirabilis* B02005 (2.5 × 10^8^ CFU/mouse), and saline water, respectively.

#### Monitoring of Animals

After infection, physiological status (body weight and fur ruffing) and feces (consistency of feces and feces water content) were observed in mice. In order to collect pathological tissues (small intestine and large intestine), animals were sacrificed for western blotting (WB), immunohistochemistry (IHC) (Xiao et al., 2015), and hematoxylin and eosin (H&E) staining (Imura et al., 2013; Zhou et al., 2018).

#### Pathological Evaluations on Mice

Pathological lesions were evaluated by double-blind test, based on the following aspects: inflammation, extent, regeneration, crypt damage, and percent involvement (Dieleman et al., 1998). Each tissue slice was graded by three investigators through the five indicators: (1) inflammation (0, none; 1, slight; 2, moderate; and 3, severe); (2) extent (0, none; 1, mucosa; 2, mucosa and submucosa; and 3, transmural); (3) regeneration (4, no tissue repair; 3, surface epithelium not intact; 2, regeneration with crypt depletion; 1, almost complete regeneration; and 0, complete regeneration or normal tissue); (4) crypt damage (0, none; 1, basal 1/3 damaged; 2, basal 2/3 damaged; 3, only surface epithelium intact; and 4, entire crypt and epithelium lost); and (5) percent involvement (0, 1–25%; 1, 26–50%; 2, 51–75%; and 3, 76–100%) (Dieleman et al., 1998; Cha et al., 2017; Qiu et al., 2017; Guo et al., 2018).

#### Immunohistochemistry

For detecting the expression of occludin protein in colon tissues, standardized method was adopted in the study (Martinez et al., 2015; Huang et al., 2018). Anti-occludin polyclonal antibody (Proteintech, United States) was mixed 1:1,000 in 5% skim milk. The sections were covered and incubated with polyclonal solutions (100 μl) at 4°C for approximately 24 h. Then the slides were washed with TBST and incubated with a secondary antibody (Dingguo Biotechnology Co., Ltd., China). The chromogenic reaction, counterstain, and dehydration were carried out; and the sections were observed by microscope (Xiao et al., 2015; Huang et al., 2016).

#### Western Blotting

Colon tissues were weighted and put into homogenizer to grind into tissue homogenate with mixture phenylmethylsulfonyl fluoride (PMSF) (*Besbio*, China) and radioimmunoprecipitation assay (RIPA) (*Besbio*, China). Protein concentration was measured by bicinchoninic acid assay (BCA) protein detection kit (*Biyuntian*, China) and micro-spectrophotometer. Proteins were electrophoresed using sodium dodecyl sulfate–polyacrylamide gel electrophoresis (SDS-PAGE) (12%) and transferred to nitrocellulose membranes. Membranes were blocked with skim milk (5%) in TBST for 1 h at room temperature and incubated with a rabbit anti-mouse polyclonal occludin antibody (diluted 1:1,000) (*Proteintech*, United States) at 4°C overnight (Zacharek et al., 2007). After being washed thrice in TBST, the membranes were reacted with a 1:1,000 dilution of horseradish peroxidase (HRP) goat anti-rabbit IgG (Dingguo Biotechnology Co., Ltd., China) for 1 h at room temperature. After being washed thrice in TBST, membranes were reacted with the mixture of peroxide solution (Bio-Rad, United States) and Luminol enhancer solution (*Bio-Rad*, United States) (1:1). Finally, the results of WB were detected by Tanon 5500 chemiluminescent imager system (*Tianneng*, China), referencing by β-actin protein as standardization. The process was repeatedly thrice for WB experiments.

### Statistical Analysis

Results were presented as mean ± SD. The data were analyzed by Student’s *T*-test and one-way ANOVA with SPSS 17.0 software. *P* < 0.05 was defined as statistically significant. ^∗^*P* < 0.05, ^∗∗^*P* < 0.01; NS, no significance.

## Results

### Bacterial Growth in Different Strains of *Proteus mirabilis*

**FIGURE 1:**
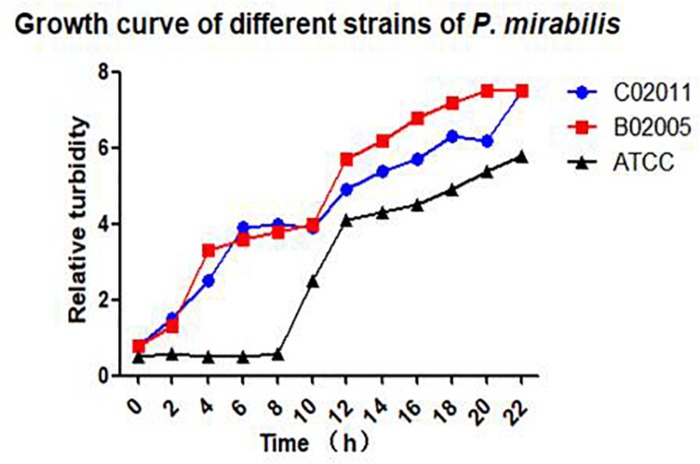
Bacterial growth in different strains of *Proteus mirabilis*. Growth curves of *P. mirabilis* C02011, B02005, and *P. mirabilis* American Type Culture Collection (ATCC) 29906 detected by Densimat photometer.

After Gram staining, morphological observations of *P. mirabilis* were examined under an optical microscope (×200 magnification). Short rod-like bacterial body stained with pink color could be observed in *P. mirabilis* C02011 and B02005 cultures. Results indicated that two isolated strains were gram-negative bacilli (see in Supplementary Appendix 1). In addition, bacterial growth was monitored by measuring optical density in cultured medium within 22 h. *P. mirabilis* C02011 strain exhibited an almost identical growth curve with *P. mirabilis* B02005 strain, whereas *P. mirabilis* American Type Culture Collection (ATCC) 29906 strain grew slower at 8 h (Figure 1).

### Caco-2 Cells May Be More Susceptible to Diarrheal *Proteus mirabilis* C02011

**FIGURE 2:**
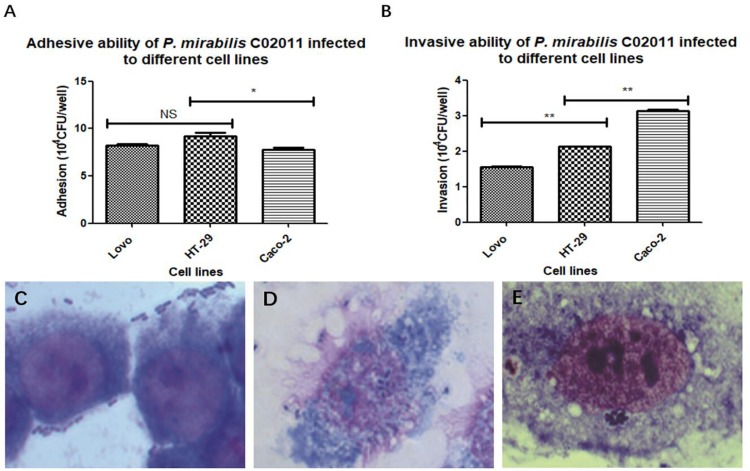
HT-29, LoVo, and Caco-2 cells were infected with *Proteus mirabilis* C02011. **(A)** The adhesion ability of *P. mirabilis* C02011 to the three kinds of cells (HT-29, Caco-2, and LoVo) was examined. **(B)** The invasion ability of *P. mirabilis* C02011 to the three kinds of cells (HT-29, Caco-2, and LoVo) were examined. **(C–E)** Giemsa staining of HT-29, LoVo, and Caco-2 cells infected with *P. mirabilis* C02011. **(C)** Giemsa staining of HT-29 cells infected with *P. mirabilis* C02011. **(D)** Giemsa staining of LoVo cells infected with *P. mirabilis* C02011. **(E)** Giemsa staining of Caco-2 cells infected with *P. mirabilis* C02011. ^∗^*P* < 0.05, ^∗∗^*P* < 0.01; NS, no significance.

In order to screen out the most sensitive cell line to diarrheal *P. mirabilis* C02011, three different types of human intestinal cell lines (HT-29, Caco-2, and LoVo) were involved in our study. Adhesion and invasion assays were used to test the bacterial susceptibility to *P. mirabilis* C02011 in human intestinal cell. Results showed that *P. mirabilis* C02011 can productively infect Caco-2 cell, compared with HT-29 and LoVo cells. By using optical microscope, we could see that large numbers of bacteria efficiently adhere to human intestinal cells (Figures 2C–E). The adhesive ability of *P. mirabilis* C02011 to the three kinds of cells (HT-29, Caco-2, and LoVo) was examined in the study; results are listed as follows: HT-29 or LoVo > Caco-2 [*P*_(HT–__29 vs. Caco–2_) = 0.014 or *P*_(LoVo vs. Caco–__2_) = 0.032, ^∗^*P* < 0.05] (Figure 2A). The invasive ability of *P. mirabilis* C02011 to the three kinds of cells (HT-29, Caco-2, and LoVo) were also examined in the study; results are listed as follows: Caco-2 > HT-29 > LoVo [*P*_(Caco–__2 vs. HT–29/LoVo)_ = 0.000, ^∗∗^*P* < 0.01] (Figure 2B). Thus, the Caco-2 cell line provides excellent *in vitro* models for evaluating the virulence of *P. mirabilis*.

### *Proteus mirabilis* C02011 Strain Is More Virulent Than *Proteus mirabilis* B02005 Strain and *Proteus mirabilis* American Type Culture Collection 29906 Strain *in vitro*

**FIGURE 3:**
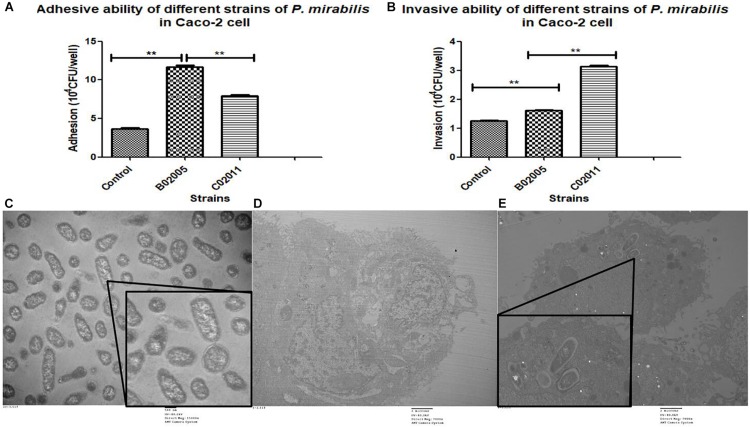
Adhesion and invasion of *Proteus mirabilis* C02011 strain, *P. mirabilis* B02005, and *P. mirabilis* American Type Culture Collection (ATCC) 29906 strains to Caco-2 cells. **(A)** Caco-2 cells were infected with *P. mirabilis* C02011, *P. mirabilis* B02005, and *P. mirabilis* ATCC 29906 strains for 2 h. The adhesion abilities of different strains were recorded. **(B)** Caco-2 cells were infected with *P. mirabilis* C02011, *P. mirabilis* B02005, and *P. mirabilis* ATCC 29906 for 2 h, followed by treatment with gentamicin (200 μg/ml) for 2 h. The invasion abilities of different strains were recorded. **(C–E)** Transmission electron microscopy (TEM) images were collected for observation. **(C)** Morphological observation of *P. mirabilis* C02011 (×15,000 magnification). **(D)** Morphological observation of uninfected Caco-2 cell (×7,000 magnification). **(E)** Morphological observation of Caco-2 cell infected with *P. mirabilis* C02011 (×7,000 magnification). ^∗∗^*P* < 0.01.

Caco-2 cells were used to evaluate the virulence of *P. mirabilis* C02011, *P. mirabilis* B02005 strain, and *P. mirabilis* ATCC 29906 strain. In the results, *P. mirabilis* B02005 strain (11.70 ± 0.36 × 10^4^ CFU/well) is more adhesive than *P. mirabilis* C02011 strain (7.90 ± 0.26 × 10^4^ CFU/well), and *P. mirabilis* ATCC 29906 strain (3.63 ± 0.32 × 10^4^ CFU/well): *P. mirabilis* B02005 > *P. mirabilis* C02011 > *P. mirabilis* ATCC 29906 [*P*_(__*B*__02005 vs. C02011)_ = 0.000, *P*_(__*B*__02005 vs._ *_*P. mirabilis*_*_ATCC 29906__)_ = 0.000, *^∗∗^P* < 0.01] (Figure 3A). On the other hand, *P. mirabilis* C02011 strain (3.13 ± 0.06 × 10^4^ CFU/well) is more invasive than *P. mirabilis* B02005 strain (1.61 ± 0.04 × 10^4^ CFU/well) and *P. mirabilis* ATCC 29906 strain (1.25 ± 0.03 × 10^4^ CFU/well): *P. mirabilis* C02011 > *P. mirabilis* B02005 > *P. mirabilis* ATCC 29906 [*P*_(__C__02011 vs. B__02005__)_ = 0.000, *P*_(C__02011 vs._ *_*P. mirabilis*_*_ATCC 29906)_ = 0.000, *^∗∗^P* < 0.01] (Figure 3B). Meanwhile, TEM images were collected to observe the infection process of *P. mirabilis* C02011 (Figures 3C–E). In Figure 3E, well-structure bacteria of *P. mirabilis* C02011 could be seen clearly in the cytoplasm of Caco-2 cells. The results of adhesion and invasion assays showed that *P. mirabilis* C02011 strain is more virulent than *P. mirabilis* B02005 and *P. mirabilis* ATCC 29906 strains *in vitro*.

### *Proteus mirabilis* C02011 Causes BALB/c Mouse Diarrhea

**FIGURE 4:**
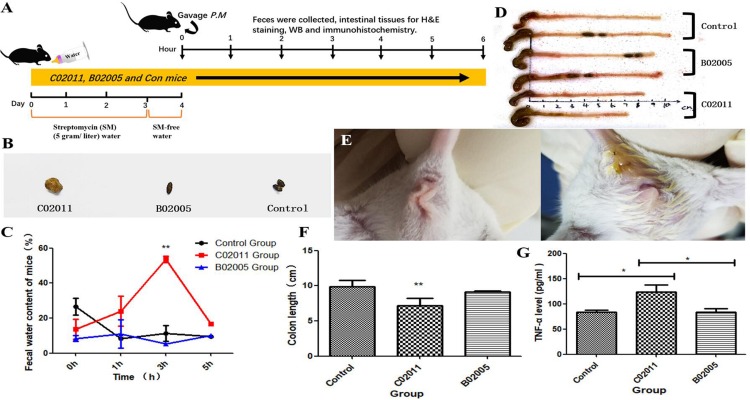
Diarrhea induced by *Proteus mirabilis* C02011 was observed in BALB/c mice. **(A)** Streptomycin-treated mice were gavaged with *P. mirabilis* C02011, *P. mirabilis* B02005, and normal saline. Clinical symptoms and colonic lesions were evaluated by indicators. **(B)** Feces images of mice in different groups. **(C)** Feces water content of mice in different groups was recorded at 0, 1, 3, and 5 h after infection. **(D)** Colon images in different groups. **(E)** Anal region images of mice in different groups. **(F)** Mouse colon length was measured in different groups. **(G)** The level of TNF-α in colon tissues. ^∗^*P* < 0.05, ^∗∗^*P* < 0.01.

SM-treated mice demonstrate increased infection susceptibility. In our study, SM-treated mice were infected with different strains of *P. mirabilis* by oral administration (Figure 4A). Then anus images, feces, and colon tissues were collected to evaluate the gastrointestinal pathogenicity induced by *P. mirabilis* C02011. After infection for 3 h, diarrhea symptoms appeared (moist anus region and loose stools) in *P. mirabilis* C02011 group mice (Figures 4B,E). Figures 4C,D show the changes in feces water content of mice in different groups. Results showed that *P. mirabilis* C02011 may cause BALB/c mouse diarrhea, although there were no clinical changes in *P. mirabilis* B02005 and control groups. In order to evaluate the colonic lesion induced by *P. mirabilis* C02011, mice were sacrificed, and their colon length was measured. After infection for 3 h, the mean length of the colons in C02011 group was significantly shorter than that of the colons in B02005 and control groups (*P* = 0.044, ^∗^*P* < 0.05) (Figures 4D,F). As we have known, the expression of TNF-α could induce the increase in Caco-2 tight junction permeability in colon tissue correlated with inflammation injuries (Song et al., 2009). Therefore, it is meaningful to detect the level of TNF-α in mouse colon tissues of different groups. Results showed that TNF-α level was significantly increased in C02011 group, compared with B02005 and control groups, at 3 h after infection [*P*_(__C__02011 vs. B02005)_ = 0.033, *P*_(__C__02011 vs. Control)_ = 0.012, *^∗^P* < 0.05] (Figure 4G). From the above results, it is reasonable to believe that mice in C02011 group suffered from more colonic lesions after infection.

### Histological Damages in the Intestine Induced by *Proteus mirabilis* C02011

**FIGURE 5:**
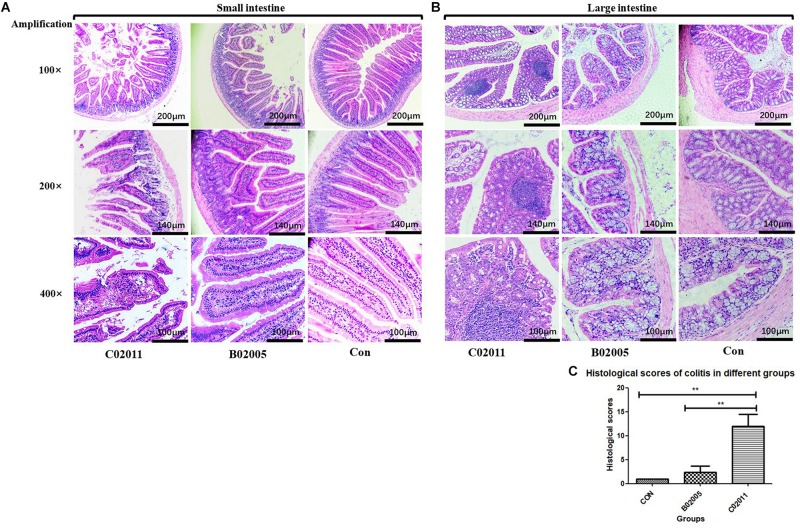
Hematoxylin and eosin (H&E) staining of the small intestine and large intestine tissues in *Proteus mirabilis* C02011, B02005, and control groups. **(A)** Histopathological observations of small bowel in *P. mirabilis* C02011, B02005, and control groups (×100, ×200, and ×400 magnification). **(B)** Histopathological observations of large bowel in *P. mirabilis* C02011, B02005, and control groups (×100, ×200, and ×400 magnification). **(C)** The average for each element in the histological score (including inflammation, extent, regeneration, crypt damage, and percent involvement) for mice in different groups. ^∗∗^*P* < 0.01.

In order to investigate the intestinal lesions induced by *P. mirabilis* in different groups’ mice, histological sections of the small intestine and large intestine were collected for histological observation (Figure 5). Under the optical microscope (×100, ×200, and ×400 magnification), lesions in the small intestine could be observed in C02011 group, whereas there were no changes in B02005 and control groups (Figure 5A). In B02005 and control groups, well-structured small intestine with regularly visible mucosa, submucosa, muscularis, and serosa could be observed. Intestinal epithelial cells were polarized in good order, whereas the nuclei were localized at the bottom of the epithelial cells. However, in C02011 group, histological damages were characterized by vacuolization of the enterocytes, swelling of the villus, destruction of the bases, and nuclei that were irregularly positioned within the cells. Few well-structured villi could be observed under the microscope. In addition, typical inflammatory lesions in the large intestine were also observed in Figure 5B. The colons collected from B02005 and control groups had normal architecture and cellular composition, whereas those of mice exposed to C02011 had edema and infiltration by inflammatory cells in the mucosal layers. Meanwhile, referring to histological grading of colitis by Dieleman et al. (1998), five indicators (inflammation, extent, regeneration, crypt damage, and percent involvement) were considered to evaluate the histological characters of the large intestine in different groups. The level of neutrophil infiltration in the colon tissues was evaluated. The scores in C02011 group were higher than those in B02005 and control groups [*P*_(__C__02011 vs. B02005)_ = 0.006, *P*_(__C__02011 vs. Control)_ = 0.002, *^∗^P* < 0.05] (Figure 5C). Histological results showed that mice in C02011 group suffered from more colonic lesions after infection.

### *Proteus mirabilis* C02011 May Induce Colonic Barrier Dysfunctions in BALB/c Mice

**FIGURE 6:**
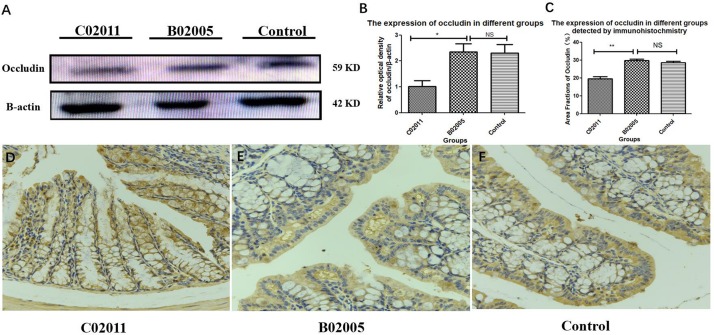
The expressions of protein (occludin) in colon tissues induced by *Proteus mirabilis*. **(A)** The expressions of protein (occludin) detected by western blotting (WB). **(B)** The occludin/β-actin intensity ratio in different groups. **(C)** The expressions of protein (occludin) in different groups detected by immunohistochemistry (IHC). **(D–F)** Large intestinal sections were stained by IHC (occludin) antibody in different groups: **(D)** IHC image in C02011 group; **(E)** IHC image in B02005 group; **(F)** IHC image in control group. ^∗^*P* < 0.05, ^∗∗^*P* < 0.01; NS, no significance.

The expressions of tight conjunction protein (occludin) declined significantly in *P. mirabilis* C02011 group, compared with *P. mirabilis* B02005 and control groups. Results suggested that *P. mirabilis* C02011 may induce colonic barrier dysfunctions in BALB/c mice (Figure 6A). WB was used in our study to detect the expressions of protein (occludin), which were related to the integrity of intestinal epithelial tight conjunction. Meanwhile, ImageJ software was applied to detect the relative optical density of protein (occludin) among three groups (Figures 6B,C). In addition, IHC was also used to directly observe the expressions of protein (occludin) in colon tissues (Figures 6D–F). From the results, the expressions of protein (occludin) in three groups were listed as follows: C02011 group < control or B02005 group [*P*_(__C__02011 vs. B02005)_ = 0.022, *P*_(__C__02011 vs. Control)_ = 0.031, *^∗^P* < 0.05]. These results indicated that *P. mirabilis* C02011 may induce colonic barrier dysfunctions in BALB/c mice.

### *Proteus mirabilis* C02011 (Wild-Type) Strain Is More Invasive Than C02011/ΔT4SS (Mutant-Type) Strain

**FIGURE 7:**
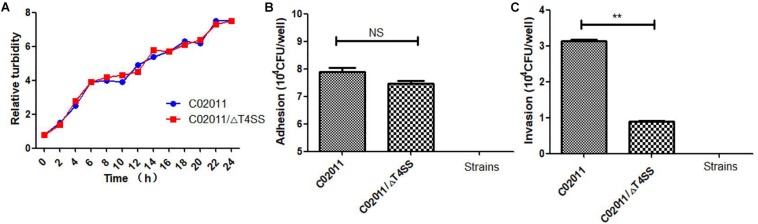
Adhesion and invasion of *Proteus mirabilis* C02011 strain and its mutant strain to Caco-2 cells. **(A)** Bacterial growth of *P. mirabilis* C02011 and its mutant strain detected by Densimat photometer. **(B)** Caco-2 cells were infected with *P. mirabilis* C02011 and its mutant strain for 2 h. The adhesion abilities of different strains were recorded. **(C)** Caco-2 cells were infected with *P. mirabilis* C02011 and its mutant strain for 2 h, followed by treatment with gentamicin (200 μg/ml) for 2 h. The invasion abilities of *different* strains were recorded. ^∗^*P* < 0.05; NS, no significance.

In our recent works, we have successfully constructed the mutant strain of *P. mirabilis* C02011 with *T4SS* genes deletion. *P. mirabilis* C02011 wild-type (WT) strain and its mutant strain C02011/ΔT4SS (MS) were involved in the experiment to preliminarily explore the role of T4SS in the pathogenesis of diarrheal *P. mirabilis*. According to the bacterial growth of two strains, we found that the WT strain exhibited an identical growth curve with the MS strain during the culture period (Figure 7A). In addition, adhesion and invasion assays were used to evaluate the virulence between two strains. Results showed that the WT strain is more adhesive than the MS strain, but with no statistical significance [*P*_(__C__02011 vs. C02011/Δ *T*4*SS)*_ = 0.070, *P* > 0.05] (Figure 7B). Invasion results indicated that the WT strain is more invasive than the MS strain [*P*_(C02011 vs. C02011/Δ *T*4*SS)*_ = 0.000, ^∗^*P* < 0.01] (Figure 7C). Hence, we preliminarily confirmed that T4SS may play an important role in the pathogenesis of diarrheal *P. mirabilis* C02011 in this food poisoning case.

## Discussion

*Proteus mirabilis* is commonly associated with urinary tract infections (UTIs) in humans, especially in patients with catheter-associated UTIs or hypo-immunity (Jacobsen et al., 2008). Some researchers suggested that *P. mirabilis* is an opportunistic pathogen (O’Hara et al., 2000; Jacobsen et al., 2008; Hola et al., 2012). However, more and more food poisoning incidents associated with *P. mirabilis* were reported worldwide (Cooper et al., 2005; Wang et al., 2010). Notably, some novel strains of *P. mirabilis* with virulence genes including *ureC*, *hpmA*, *qnrD*, and *T4SS* have been successfully isolated from food poisoning specimens. Hence, it is meaningful to determine the gastrointestinal pathogenicity of these novel strains and explore the mechanism.

As we known, LoVo, Caco-2, and HT-29 cell lines are commonly used as *in vitro* models for elucidating the pathogenic mechanism in the intestine (Bu et al., 2011; Qiu et al., 2016). A previous study showed that Caco-2 cells could be used as a sensitive model for investigating the function of intestinal barrier (Bu et al., 2011). Therefore, three colon cancer cell lines were involved in our study. In order to build a cell model of infection, adhesion and invasion assays were performed to evaluate the bacterial susceptibility of diarrheal *P. mirabilis* to three human intestinal cell lines (Kim and Wei, 2007; Qiu et al., 2016; Yang et al., 2017). Moreover, MOI (MOI = 100) and optimal time for infection (optimal infection time = 2 h) have also been explored in our preliminary experiments. After that, qualitative assays (Giemsa staining and TEM) and quantitative assays (adhesion and invasion) were performed to better evaluate the virulence of *P. mirabilis* strains.

On the other hand, BALB/c mice were used to build the diarrhea animal model for *P. mirabilis* C02011. In order to simulate the infective route of patient in food poisoning case, mice were challenged with *P. mirabilis* C02011 and B02005 by oral administration. However, there is one problem: It is not easy to build a diarrheal mouse model induced by intestinal pathogen because of colonization resistance (intestinal microflora) (Kim et al., 2017). In our previous works, we have made a substantial effort to make a sensitive mouse model induced by *P. mirabilis*. Furthermore, we have explored oral doses of *P. mirabilis* (10^7^, 10^8^, 10^9^, and 10^10^ CFU/ml), optimal culture medium of *P. mirabilis* (Luria broth and BHI), and culture temperature of *P. mirabilis* (25°C and 37°C). Nevertheless, diarrhea could not be observed in an animal model infected with *P. mirabilis.* In order to solve this problem, antibiotic pretreatment has been used to reduce the colonization resistance. In 2006, Suar and his team successfully built an SM-pretreated mouse model of *Salmonella* by oral cavity infection with typical symptoms of intestinal injury (Suar et al., 2006). Coincidently, in 2016, Elder et al. (2016) had also built a mouse model of *Salmonella* with inflammatory diarrhea by SM pretreatment. In 2018, Mullineaux-Sanders et al. (2018) published their work in *Nature Microbiology* and concluded that commensal colonization may be the reason for inhibiting enterohemorrhagic *Escherichia coli* (EHEC)- and *Salmonella*-induced colitis in a mouse model, which could be effectively improved by SM pretreatment (Kim et al., 2017; Mullineaux-Sanders et al., 2018).

In the study, the gastrointestinal pathogenicity of *P. mirabilis* C02011 isolated from a food poisoning case has been explored for the first time. Moreover, we successfully established the diarrheal BALB/c mouse model induced by *P. mirabilis*. Both *in vitro* and *in vivo*, we successfully validated that the novel strain of *P. mirabilis* C02011 has more diarrheagenic virulence than have other strains isolated from healthy individual. Notably, *T4SS* genes were identified in *P. mirabilis* C02011 by whole genome sequencing, which were not found in *P. mirabilis* B02005. On the next step, C02011/ΔT4SS, C02011/ΔVirB9 (an important structural protein in T4SS transmembrane complex) (Jakubowski et al., 2005), C02011/ΔVirB11 (a subunit of T4SS related to ATPase) (Smith et al., 2016), and their complemented strains will be built for the further explorations on the pathogenic mechanism of *P. mirabilis* C02011.

However, several issues need to be addressed in our study. Firstly, T4SS was considered as the reason for causing diarrhea induced by *P. mirabilis* C02011. But in the study, we have not yet explored the function of T4SS and its subunits (VirB9 and VirB11) completely. Secondly, *P. mirabilis* C02011 involved in the study was isolated from patients’ specimens in a food poisoning case. It is less convincing to completely exclude the possibility whether diarrhea disease was caused by host low immunity or bacterial virulence, so it is essential to determine the bacterial pathogenicity both *in vitro* and *in vivo*.

## Data Availability Statement

The datasets analyzed in this manuscript are not publicly available. Requests to access the datasets should be directed to 1143115967@qq.com.

## Ethics Statement

The animal study was reviewed and approved by the Animal Care Committee of Southern Medical University (Guangzhou, China).

## Author Contributions

HC, XS, QH, and ZG designed the whole experiments. ZG and HC wrote the manuscript. ZG, FB, HZ, YuL, and YW did the animal experiments and cell experiments. XH, YiL, YQ, and QC did the technical guidance.

## Conflict of Interest

The authors declare that the research was conducted in the absence of any commercial or financial relationships that could be construed as a potential conflict of interest.
