# Supplementary figures and images for: Characterization of a Novel Diarrheagenic Strain of Proteus mirabilis Associated With Food Poisoning in China
Source: Front Microbiol. 2019 Dec 12;10:2810. doi: 10.3389/fmicb.2019.02810 (PMC6921692; doi:10.3389/fmicb.2019.02810)

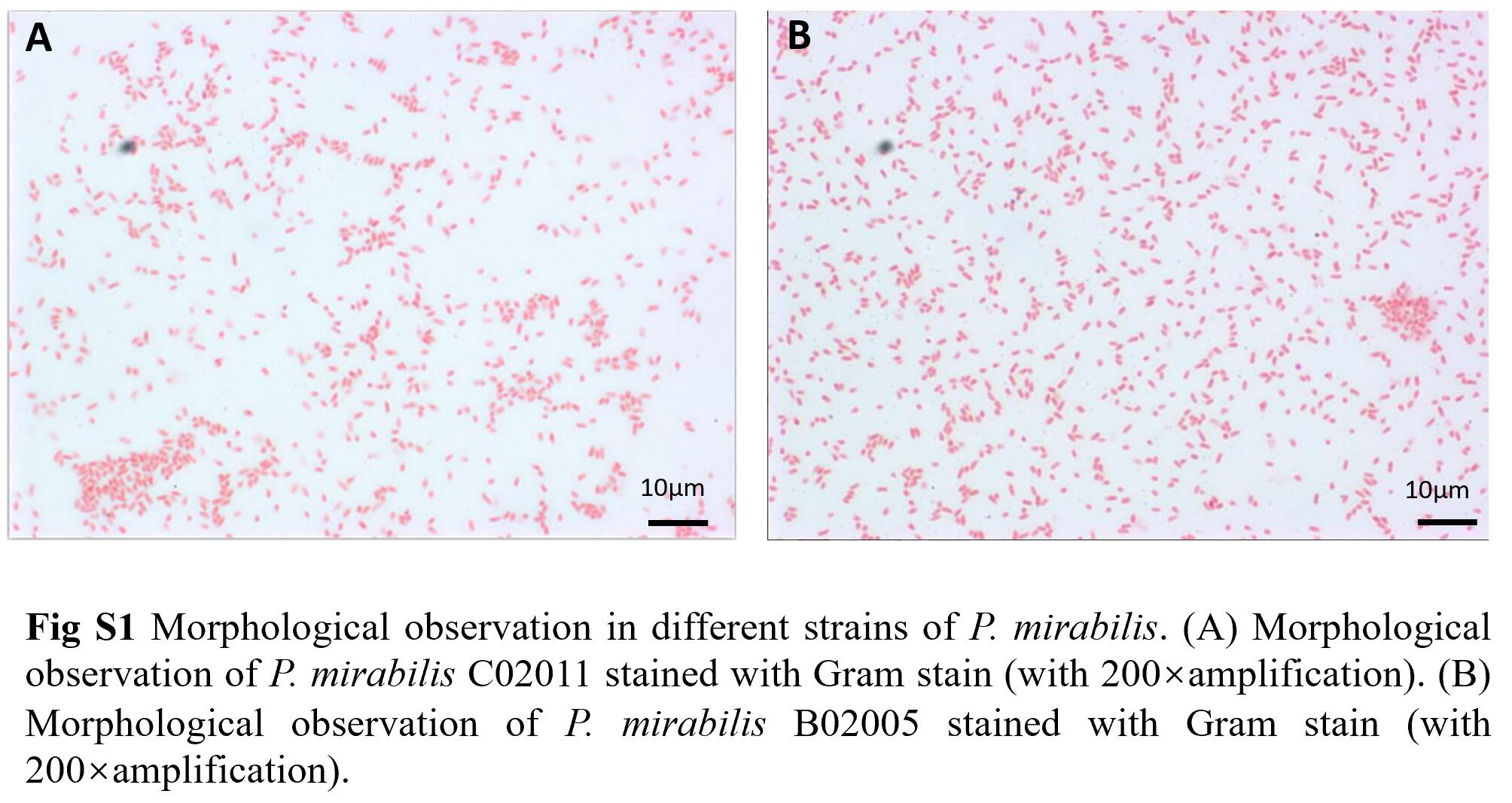

Supplement: Supplementary file 1 [file Image_1.jpg]

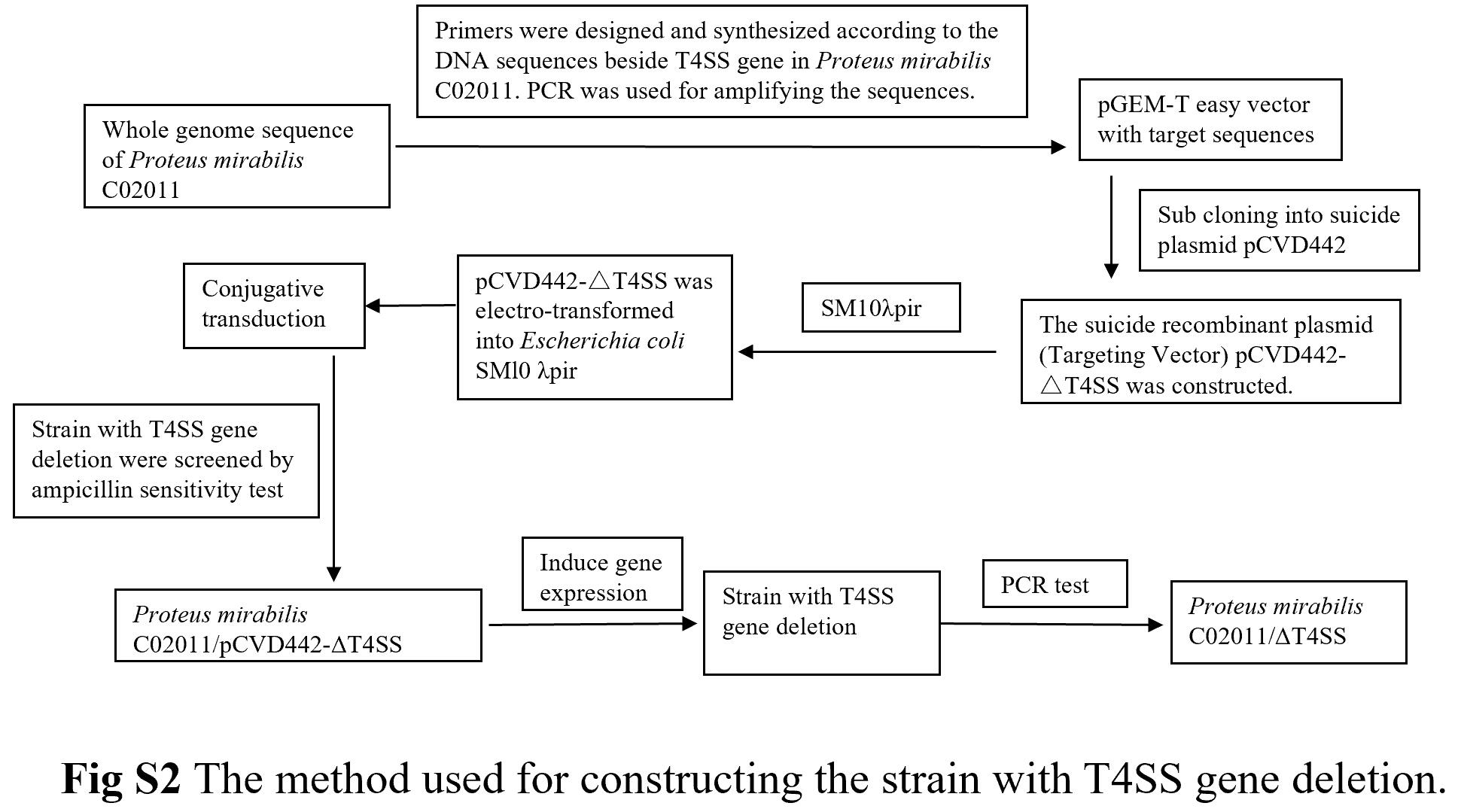

Supplement: Supplementary file 2 [file Image_2.jpg]
